# On the Effects of Opium on the Infant Subject

**Published:** 1844-02

**Authors:** John B. Beck

**Affiliations:** Professor of Materia Medica, &c., in the Col. Phys. and Surg., N. Y.


*On the Effects of Opium on the Infant Subject.* By John B. Beck, M.D., Professor of Materia Medica, &c., in the Col. Phys. and Surg., N. Y. The following practical observations are extracted from a paper in the January number of the New York Journal of Medicine. The subject is one of much concern to the profession and the public:

"With regard to the effects of opium on young subjects, there are two facts which seem to be well established. The *first* is, that it acts with much *greater energy* on the infant than it does on the adult; the *second* is, that it is more *uncertain* in its action on the infant than the adult. It is in consequence of these peculiarities attending its operation on the infant, that even the smallest quantities have not unfrequently produced the most unexpected and even fatal results. Of this, almost every physician must have seen some melancholy instances. Dr. John Clarke states, that 'half a drachm of genuine syrup of white poppy, and, in some instances, a few drops of Dalby's Carminative, have proved fatal, in the course of a few hours, to very young infants.' In one case, he says, forty drops of Dalby's Carminative destroyed an infant. Mr. Marley says, 'I have known three or four instances where the most dangerous symptoms were produced by Godfrey's Cordial and Dalby's Carminative; two nostrums which have no doubt added considerably to the mortality of infants.' In a case that fell under his observation, the most rapid and alarming symptoms followed the exhibition of an ordinary dose of syrup of poppies. In another case, he knew half a small teaspoonful of the syrup of poppies prove nearly fatal to a child eight or ten days old. Thirty-five drops of Dalby's Carminative, he has known to prove fatal to a young child, while, in other cases, larger doses have been given without any unpleasant effects. The same writer relates the case of an infant, nearly poisoned, by considerably less than half an ordinary-sized tea-spoonful of paregoric. Dr. Bard says he once knew an infant of several months old killed by ten drops of laudanum, and another brought into very great danger by less than two drops. Dr. Montgomery states that he has known more than one instance in which a tea-spoonful of the syrup of poppies has proved fatal to a healthy child. Professor Hamilton relates two cases, in which four drops of laudanum proved fatal to children some months old. Dr. Merriman reports two cases, in which a dose of Godfrey's Cordial proved fatal. He also states that he once saw a child in the month thrown into a state of excessive stupor, by taking one dose only of a mixture in which there were four drops of laudanum; the actual quantity swallowed could scarcely have amounted to one drop. Dr. Christison states that 'the administration of three drops of laudanum in a chalk mixture for diarrhœa, to a stout child, fourteen months old, was followed by coma, convulsions, and death in six hours.' In another infant, a few weeks old, death resulted from taking four drops of laudanum. Dr. Ryan states that he has known one drop of the 'sedative liquor of opium' narcotize an infant. Of laudanum, two drops have been known to kill an infant, nay, in one case, a single drop destroyed a new-born infant. I have myself seen a young child narcotized by about twenty drops of paregoric. The foregoing facts are sufficient to show that opium acts with peculiar energy and uncertainty upon the infant subject. The causes of this would seem to be the following:

In the *first place*, the great difference in the physical organization of the *infant* and the *adult*. In the young subject the brain and nervous system are much more impressible, and the slightest causes, as we know, will sometimes derange them. Besides, in the infant, the circulation is more rapid—there is a greater proportionate quantity of blood circulating in the brain, and hence a much greater tendency to cerebral determinations. From these peculiarities in the organization of the infant, it happens that convulsions are so much more common in the early periods of life. Thus, for example, the irritation of teething—of worms or crude matters in the intestines, is frequently followed by convulsions. Intermittent fever, which in the adult commences with a chill, in the child is frequently ushered in by a convulsion. Scarlet fever, too, in the child, not unfrequently commences with a convulsion, while in the adult I have never witnessed such an occurrence. Now, with such peculiar predispositions characterizing the system in infancy, it may readily be conceived how it is that such an article as opium should act with more power at that period than in after life.

"In the *second place*, the difference in the *temperament* or *constitution* of infants. In the adult, we know as a matter of fact, that opium differs greatly in its effects in different constitutions. Thus, as a general rule, the sanguine temperament does not appear to bear the use of this drug as well as the melancholic or the nervous. In the former, it is much more apt to produce cerebral disturbance, and in large doses is more likely to prove injurious. Now, infants differ from one another as much, if not more, than adults, in these peculiarities of constitution, and, as a matter of course, the difference in the effects of this article must be greater. Besides, as these peculiarities and differences can only be detected by actual experience, and as we cannot of necessity have the same benefit of experience in the case of infants, it is obvious that the difficulty of justly appreciating the action of this drug on the infant must be greatly increased. A greater or less degree of uncertainty, therefore, must necessarily from this cause attend its use in the early periods of life.

"In the *third place*, the actual state of the system as to disease. There is no circumstance which modifies the effect of opium in so great a degree as this. In the adult, we see this continually. In some conditions of the system, even small doses produce the most unpleasant effects, while in other conditions, immense quantities may be given with little or no effect; thus, for example, when severe pain or spasm are present, quantities of this article can be borne, which under other circumstances would prove exceedingly injurious. As illustrative of this, I quote an intesesting case related by Dr. Percival. He states that a young man was admittted into the Manchester Hospital, on account of a violent spasmodic disease which recurred periodically in the evening, and after trials of various remedies, doses of opium sufficiently large to mitigate the violence of the paroxysm were ordered, and he took twenty-two grains every night during a week, without producing any soporific effect. On the eighth night he had no return of the spasm. He nevertheless took the opium, and in the morning was found dead. In this case, a great and sudden change had unquestionably taken place in the nervous system of the patient, and to this must be ascribed the difference of effect. If in the adult, the state of the system makes such a wide difference in the effects of this article, how much more so must all this be the case in the sensitive infant; and it is by not duly regarding this, that such unexpected results sometimes follow from the use of opium. Thus, for example, a child laboring under the acute pain of colic will tolerate doses, which, in the ordinary condition of the system, might prove destructive.

"There is one condition of the young subject particularly in which this remedy is frequently resorted to, in which this is strikingly illustrated. I mean that state of exhaustion which arises from diarrhœa and other bowel complaints. In this state the head is very apt to become affected, and if opium be given with a view of checking the intestinal discharges, not unfrequently insensibility gradually creeps over the little sufferer, and in a short time death is the result; and this, too, even when the quantity used has been apparently adapted with great nicety to the wants of the case. Every observing practitioner must have witnessed such instances. Now in many cases of this kind, there can be no question that the child sinks under the sedative influence of the opium; and the reason is, that, in the exhausted state brought on by disease, the system succumbs much more readily to the narcotic effects of this article, than it does in other conditions of the system.

"The foregoing considerations appear to me sufficient to account for the greater power, as well as uncertainty in the action of opium on the infant, than on the adult.

"If it be a fact, then, that opium acts in this way upon the infant, it appears to me to involve inferences of great practical moment, which cannot be too deeply impressed on the mind of the young practitioner.

"1. That its use should *be avoided as much as possible* in the young subject. I will not say that it ought to be interdicted altogether, because if used with discretion, it is a remedy of great value in many of the diseases of infants, but it should never be used unless there exists a strong and manifest necessity for it.

"2. Great caution should be exercised in the form in which it is administered. No preparation should ever be used, which *is not of a known and determined strength.* In England, the *Syrup of Poppies* is the preparation most used for children. In this country, it is also used, but not to the same extent. This is a pleasant and mild opiate, and on these accounts is well adapted for children. It is liable, however, to great objections. Besides, being apt to ferment and spoil, it is very *variable in its strength.* On this account it is really a very dangerons article; and many cases are recorded (some of which I have already related) in which fatal results have followed the use of it, even in moderate doses. Another objection is, that it is liable to sophistication. Thus a mixture of *laudanum and simple syrup* has sometimes been sold for it. In the London Medical Gazette, (May, 1831, p. 253), a case is related, where a child died in consequence of a small dose of this latter compound having been given by the mother, who had previously given the same quantity of the pure Syrup of Poppies with advantage.

"The best preparations for children are *laudanum* and *elixir paregoric.* These are of known strength, and susceptible of division into the minutest doses. *Dover's powder* is another preparation, which may be given to children. It may readily be divided into the smallest doses, and it seems to act much more mildly than equivalent doses of simple opium. It need hardly be stated that all such articles as Godfrey's Cordial, Dalby's Carminative, &c., should be totally discarded from regular practice. Besides being uncertain in their strength, and on that account exceedingly objectionable, the sanction thus given to them encourages their use by persons out of the profession, who cannot be supposed to be acquainted with the dangerous effects of opium on the infant system.

"3. In very young subjects, we should never begin the use of this article, except in *very small doses.* Although most practical writers lay down cautions about the use of opium in these cases, yet it does not appear to me that these cautions are sufficiently precise. Most of the writers to whom I allude, specify doses as suitable to certain ages, without stating, that even these doses may, in certain conditions of the system, prove just as injurious as much larger doses. To illustrate, I will quote the directions given by one of our standard authorities. Dr. Dewees says, 'the proper dose of laudanum for infants and children may be reckoned at the following rates. Half a drop for a child under ten days old; a drop, for one from that period to the end of the month; a drop and a half, or two drops, for one from that period to three months; three drops from this to nine months, &c., &c.' 'When laudanum is to be used as an injection, we may safely increase the quantity three or four fold' He adds: 'These doses are prescribed for children who are altogether unused to the use of this drug; the power of bearing more, may be rapidly increased by habit.' Now, it appears to me that a more dangerous set of directions could not well have been given. Although many children may bear the quantities here specified without injury, yet evey now and then a case will occur, in which the most serious results will follow; and it is against these that the necessary precautions should always be taken. In the case of a new-born infant, we are entirely ignorant of the manner in which such an article as this will affect it, and it therefore will not do to begin with *average* doses. To practise safely, we must feel our way with doses much smaller; and then we shall have some guide, and the only guide which the nature of the case admits of, to make the necessary increase in the quantity to be given. Under no circumstances, as a first dose, ought half a drop to be given to a child under ten days—or a drop to a child during the first month. One eighth of a drop is sufficient to begin with. The quantity, too, directed for an injection is too large. Instead of three or four times the quantity given by the mouth, as far as my experience goes, double the quantity is quite sufficient.

"4. The doses of opium should not be repeated *at too short intervals.* This, too, is a point which is not sufficiently guarded by some practical writers. One writer, for example, after specifying the quantity suitable for a child of two and three months, adds, that 'this is not to be repeated in less than an hour.' If this means anything, it means, of course, that after the lapse of an hour, the dose may be repeated with safety. This, however, will not be sustained by experience. Even if a first dose does not narcotize, it frequently produces a degree of listlessness and indifference to food on the part of the child, which, if it be kept up by repetitions of the opiate, may eventually prove just as destructive. This is strikingly illustrated in those states of exhaustion from diarrhœa, where the due supply of nourishment is so essential to recovery. Where repeated opiates are necessary, the intervals between the doses should be long enough to enable the child to recover somewhat from the sedative influence.

"Before concluding these observations, I cannot refrain from making a remark or two in relation to the use of this article by persons out of the profession. The mischief that is done in this way is incalculable. If, in the hands of those acquainted with its virtues, opium is an article so dangerous and uncertain in its action, what must it be in the hands of the ignorant; and yet we see it given to infants, day after day, and night after night, by nurses and mothers, not merely without the consent of the physician, but sometimes contraay to his express injunctions.

"There are two ways in which it is used by persons out of the profession, in both of which it proves injurious to the child. The first is by giving it in occasional doses; the second, by giving it constantly. The first is bad enough, but the second is still worse. The first, now and then, unexpectedly destroys a child; the second is followed by a train of the most disgusting consequences, worse, if possible, than those of habitual drunkenness in the adult. Fortunately, these latter cases are not of such frequent occurrence; occasionally, however, they are met with where the parent, for the purpose of quieting it, has been induced to keep a child for months under the daily influence of paregoric, Godfrey's Cordial, or some other opiate nostrum. In these cases, the effect is to stunt the growth of the child; it is emaciated and puny; the skin is flabby and shrivelled; the lips are bloated, and the countenance sallow and wrinkled. There is an absence of all intelligence, and the whole appearance is haggard and aged, presenting a sort of 'miniature of old age.' Not long since I witnessed a case of this kind, in which a child of fourteen months old did not appear larger than one of two or three months. With the exception of one month, it had been kept upon paregoric almost every day since its birth. The mother was a poor woman, and on inquiring of her the reason, she stated that she had resorted to this method of keeping the child quiet while she attended to her work.

"Of the extent of the mischief annually perpetrated by the unprofessional use of opium, some idea may be formed from a report made to the House of Commons, containing returns from the coroners of England and Wales, of the inquisitions held by them during the years 1837 and 1838, in cases of death by poison. The total number of deaths by poison in these years was 543, of which 52 were very young children, most of them at the breast, in conseqgence of opium, or some of its preparations having been given by mothers and nurses, in ignorance of its effects. In addition to this, 20 more were destroyed by opium or laudanum administered in mistake for other medicines.

"These facts are certainly appalling; and if any subject connected with medical police is worthy of the atttention of the public authorities, it is certainly this. How the evil is to be corrected, it is not easy to say. Much, however, might be done by the proper dissemination of information on the subject. In most cases where opium is administered to children by persons out of the profession, it is disguised in the shape of some nostrum, so that they are not aware of what they are giving, and even when they are aware of it, they are not acquainted with the dangerous effects of it on the infant system. If parents and nurses were made better acquainted with the fact that such articles as Godfrey's Cordial, Dalby's Carminative, and most of our medicated lozenges and candies, owe all their composing properties to the opium which they contain, and that opium even in small doses is frequently a deadly poison to the infant, one would suppose that it could not but exert a salutary influence in correcting, to a certain extent at least, the evil of which we are speaking, and the dissemination of this kind of knowledge by the proper authorities would confer a lasting benefit upon the community.

"I have thrown together the preceding observations because the subject is one of interest and importance, and because it appears to me that it has not sufficiently attracted the attention of the profession."

